# Preschool teachers’ perspective on how high noise levels at preschool affect children’s behavior

**DOI:** 10.1371/journal.pone.0214464

**Published:** 2019-03-28

**Authors:** Kerstin Persson Waye, Sofie Fredriksson, Laith Hussain-Alkhateeb, Johanna Gustafsson, Irene van Kamp

**Affiliations:** 1 Occupational and Environmental Medicine, Public Health and Community Medicine, The Sahlgrenska Academy, University of Gothenburg, Gothenburg, Sweden; 2 Swedish Institute for Disability Research (SIDR), School of Health Sciences, Örebro University, Örebro, Sweden; 3 Centre for Sustainability, Environment and Health, National Institute for Public Health and the Environment (RIVM), Bilthoven, The Netherlands; University of Wuerzburg, GERMANY

## Abstract

Early-age exposure to noise may have long-term health implications of which we have little knowledge of today. Age-specific hearing, learning inadequate coping strategies, and alterations in biological stress regulatory responses could play a role in the long-term health impacts. In Sweden about half a million children in the age between 1–5 years attend preschool. The noise exposure at preschools is intermittent and unpredictable and levels reach up to 84 dB LAeq (time indoors) with maximum levels of 118 dB LA_F_, mostly due to child activity. To increase the overall understanding of the possible implications of preschool noise environments for children, this paper describes children’s behavioral and emotional reactions to and coping with their everyday sound environment from a teachers perspective. A postal questionnaire study performed in 2013–2014 with answers from 3,986 preschool teachers provided the data. Content analysis was combined with quantitative analysis. Eighty-two percent of the personnel considered that children’s behavior was affected rather or very much by preschool noise. The most prevalent behaviors were categorized into: be heard, be distracted, show negative internal emotions, crowd, avoid, withdraw, be exhausted, and learning. The quantitative analyses confirmed an association between the perceived loudness and noise annoyance at preschool and affirmative reporting on noise affecting the children´s behavior. Age of the personnel, with the youngest age group reporting noise related behavior less often, and age distribution of the class, with 1–5 years old seeming less affected by noise, were also indicated, while pedagogic orientation was not a significant factor. Future studies should address the long-term health effects of these behaviors.

## Introduction

In Sweden, more than 80% of 1–5 year old children spend most of their day in preschool. They are exposed to high levels of intermittent and often high frequency sounds. Children’s voices and activities are the main noise sources. Earlier measurements show that preschool children are on average exposed to of 84 dB LAeq_Time indoors_, with maximum noise levels reaching LAFmax 118 dB [1]. These levels exceed the Swedish Work Authorities limits [2], which are aimed to reduce the risk of hearing impairment caused by occupational noise. A large proportion of preschool teachers report exposure to high sound levels and noise annoyance. They also report emotional stress and have a higher risk of hearing-related symptoms compared to individuals in other occupations [3, 4], but little is known on how preschool children are affected. Groundbreaking knowledge on children’s hearing [5], showed that the diffraction and reflection properties of the head, pinna and torso, the Head Related Transfer Functions (HRTF) from children are not comparable to an adult’s. In short, this means that a child below the age of about seven will receive frequencies of around 6 kHz being substantially more amplified, as compared to a young adult and adult whose amplification is strongest around 3kHz. Today it is not known if this amplification increases the risk for hearing disorders, though we can postulate that certain high frequency sounds originating from contact between surfaces such as clatter of cutlery and plates against a tabletop, and chairs being pulled over the floor may be perceived as particularly unpleasant. In addition, children could experience other children’s high frequency pitch screaming as especially painful since they have potentially more sensitive high frequency hearing.

In earlier qualitative studies among 4–5 year old preschool children, it was shown that children described their sound environment in a varied way [6]. Their descriptions were categorized into: trustful sounds (everyday sounds from known trusted individuals), neutral sounds (from washing machines), unknown frustrating sounds (sounds from the radiator), and distressing sounds. Distressing sounds were described as painful (high frequency and sudden sounds like screeching sounds from the swing and rakes, screaming from other children), and threatening sounds (situations involving screaming of specific children who often initiated violent situations). A model was developed and formulated as “Living with own uncontrollability of sounds and noise”. It was further found that children described coping with their sound environment by going away, hiding, cover their ears, but also sometimes expressing “not knowing what to do”. The coping strategies were later confirmed in an intervention study where more than 70% of the children adopted some coping strategy when exposed to loud or unpleasant preschool noise [7].

To increase the overall understanding of the possible implications for children of being in a preschool noise environment, this paper describes the personnel perspective on how high noise levels at preschool may affect children’s behavior. The data was analyzed using content analyses of manifest contents combined with a sensitivity analysis of a random sample of personnel reports. To provide a contextual framework for the content analyses we performed a quantitative analysis exploring the effect of noise exposure, pedagogic orientation of the preschool, and age of the preschool teachers for the odds ratio (OR) of teachers reporting noise to influence the children’s behavior.

## Methods

### Source material

The current analysis is based on data from a postal questionnaire study performed in 2013–2014. The aim of the initial study was to evaluate how current and previous occupational sound environment affected hearing and health among preschool teachers in comparison to a randomly selected population of women [3]. Initially, 11,232 men and women possessing preschool teachers’ degrees were sent a 56-item questionnaire, and 5,687 responded (51%). After excluding all male preschool teachers (*n* = 168), the female respondents who had never worked in preschool (*n* = 489) and the female preschool teachers who had early retirements or yet working but above the retirement age of 65 (*n* = 312), a total of 4,718 preschool teachers were summed with questionnaire responses. Out of these, 3,986 preschool teachers responded to the filtering question: “Do you find that preschool noise affects the behavior of the children?” (Q52) and those with missing on this key question were excluded. The age range among the 3,986 preschool teachers included in the analysis was 24 to 65 years, with a mean age of 44.3 years (standard deviation, SD: 9.7). All had more than 3 years of university education and a majority of them, 91% (*n =* 3,631), were currently working. The rest were either on short-term parental leave or leave of absence from work (5%, *n =* 189), or unemployed, studying, on sick leave or ‘other’ (4%, *n =* 163), basing their responses on previous experience from working in preschool.

The response alternatives for Q52 were: not at all, somewhat, rather much and very much. If responding at least ‘somewhat’ to Q52, subjects were encouraged to describe how, in their own opinion, the noise affects the children, using free text in an open-ended question. These free-text responses were later analyzed qualitatively. The question was first developed in a previous study investigating the effect of an intervention in preschools [1].

The regional ethics committee in Gothenburg approved this study (Dnr 060–13).

### Quantitative analyses

For the quantitative analysis, explanatory factors were derived from questionnaire items describing loudness and annoyance of occupational noise exposure (three items), preschool related factors (two items), and age of respondents (one item). They were assessed in relation to the key question: “Do you think that preschool noise affects the behavior of the children?” (Q52). This key question was assessed as the dependent outcome variable, with responses “not at all” and “somewhat” classified as 0, and “rather much” and “very much” as 1. The items used to assess noise exposure were: 1) “Is the sound level so loud that you have difficulties hearing what other people say?” (Q6) 2) “How often are you exposed to sound levels so loud that you must raise your voice to be heard?” (Q7). Q6 and Q7 were answered on a 5-category scale from never, about 25% of the time, about 50% or the time, about 75% of the time, and always/almost always. 3) “Are you at your current workplace annoyed by sound/noise?” (Q5). Answers were given on a 5-category scale from not at all, somewhat, rather much, very much, and extremely much. Preschool related factors were: 1) “What is the educational orientation of your preschool?” (Q52): Unspecified public preschool, Outdoor education (Ur & Skur), Montessori, Waldorf, or other. 2) “What are the age groups in the classes at your preschool?” (Q50), 1-5years, 1–3 years, 3–5 years, or other. As for individual factors of the personnel, age was assessed as an explaining variable in the analysis as age increases the risk for hearing related outcomes such as hearing loss and tinnitus, which could affect how the teachers perceived the sound and the behavior of the children. The quantitative analysis was performed using logistic regression; the explanatory factors were included as independent variables and the odds ratio (OR) for each response category was estimated in comparison to a reference response category. In a crude analysis, only one factor at a time was included in the model, while the final adjusted model included all items simultaneously. The likelihood ratio test was used to assess model fit. A significance level of p<0.05 was applied. Education (preschool teacher’s degree) and sex were part of the inclusion criteria.

### Content analyses

The free text was analyzed in a content analysis using the software Open Code 4.03 (OpenCode 3.4. Umeå University 2013). In performing content analyses, one may focus on the manifest content or the latent content. A manifest analysis describes the visible, obvious components, what the text says, while a latent analysis tries to analyze the underlying meaning of the text [8]. They both involve interpretation, but varies in depth and level of abstraction [9]. In this article, a manifest analysis of the free text given by the personnel was initially carried out, with an inductive category development. The unit of analysis for the coding included the whole text given by each personnel, usually comprising around 10–20 words.

The procedure of the manifest coding was initiated by reading through all the text to achieve an overall picture of the context. Frequently occurring words or concepts were written down, and these word stems e.g. focus were searched using the software Open Code. The results recorded the number of free texts or responses where the stem focus e.g. focused, focusing, out of focus was included. The word stems were added step by step to categories or themes and during the process revised and reformulated and labeled to achieve a condensed common meaning. The categories were derived to share an internally homogeneous and external heterogeneous commonality in accordance with [10]. An intra-rater comparison was made by randomly selecting 50 samples of the free text, and with one of the authors separately reading them through in detail to look for additional categories. No new categories emerged. In the process of analyzing the data, on-going comparisons were made where all the authors discussed the codes and categories.

## Results

**Fig 1:**
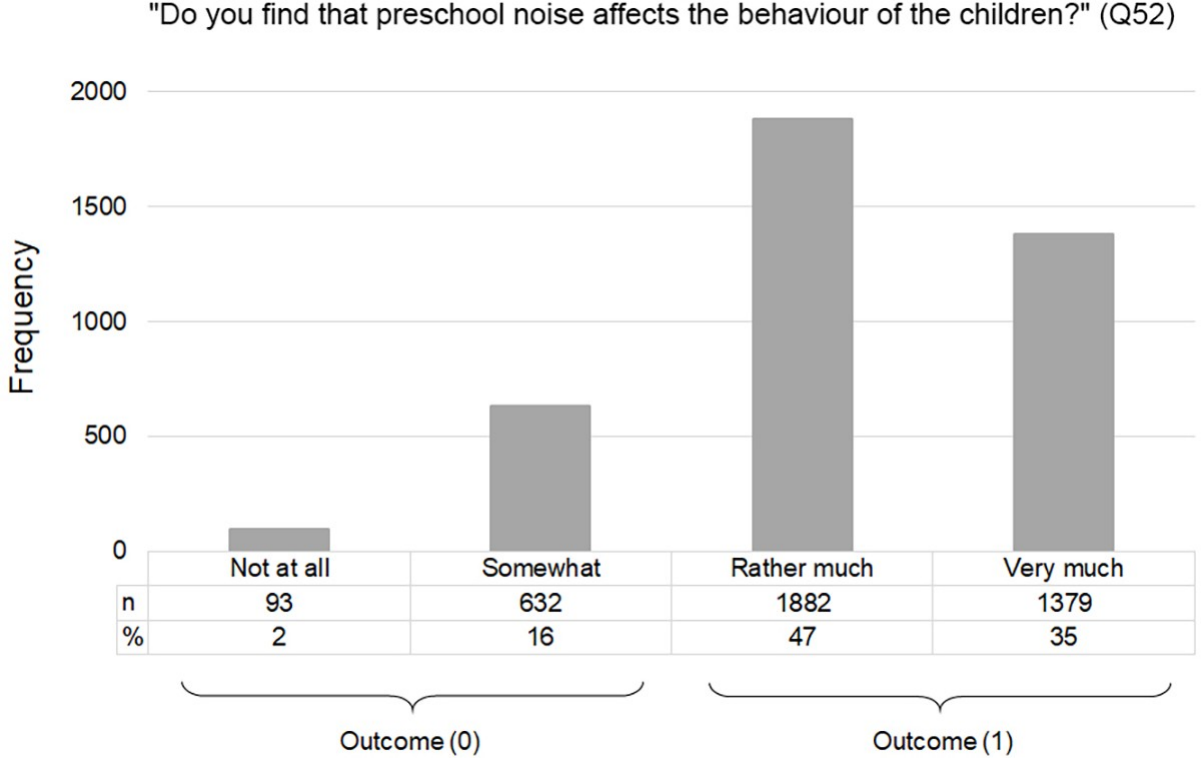
Number (n) and proportion of respondents for each response alternative to the key question: “Do you think that preschool noise affects the behavior of the children?” (Q52), and the definition of the outcome variable.

Fig 1 shows the distribution of the outcome variable. The large majority of the preschool teachers, 82%, reported that preschool noise affects children’s behavior rather much or very much.

### Quantitative analysis

The odds ratio (OR) for the outcome, modeled in relation to explanatory factors, are given in Table 1. According to the likelihood ratio test the presented model had the best fit explaining 13.8% of the outcome.

**Table 1:** Logistic regression showing odds ratio (OR) with 95% confidence interval (CI) for the dependent binary outcome based on the key question: “Do you find that preschool noise affects the behavior of the children?” (Q52), related to single explanatory variables (crude analysis) and with adjustment to other explanatory variables (adjusted analysis). Odds ratio of 1 denotes equal to the reference category, an OR < 1 denotes lower than the reference category and an OR > 1 denotes a value higher than the reference category.

Explanatory variables (n responses)	Crude analysis		Adjusted analysis*	
	OR (95% CI)	p-value	OR (95% CI)	p-value
Q6) Is the sound level sometimes so loud that you have difficulties hearing what other people say?				
Never (n 815)	ref.		ref.	
about 25% of the time (n 1,528)	1.9 (1.5 to 2.3)	0.001	1.3 (0.9 to 1.7)	0.121
about 50% of the time (n 881)	4.1 (3.2 to 5.4)	0.001	1.5 (1.0 to 2.3)	0.053
about 75% of the time (n 467)	4.8 (3.4 to 6.9)	0.001	0.9 (0.5 to 1.5)	0.675
Always/almost always (n 145)	22.8 (7.2 to 72.2)	0.001	2.7 (0.7 to 10.1)	0.131
Q7) How often are you exposed to sound levels so loud that you must raise your voice to be heard?				
Never (n 806)	ref.		ref.	
about 25% of the time (n 1,504)	1.9 (1.5 to 2.3)	0.001	1.5 (1.1 to 2.1)	0.005
about 50% of the time (n 907)	3.4 (2.6 to 4.3)	0.001	1.9 (1.3 to 2.8)	0.002
about 75% of the time (n 448)	9.3 (5.9 to 14.7)	0.001	5.2 (2.8 to 9.8)	0.001
Always/almost always (n 174)	11.5 (5.3 to 24.9)	0.001	3.6 (1.4 to 9.2)	0.006
Q5) Are you annoyed by noise at your workplace?				
Not at all (n 196)	ref.		ref.	
Somewhat (n 799)	0.5 (0.3 to 0.7)	0.001	0.8 (0.5 to 1.3)	0.314
Rather much (n 1,134)	1.1 (0.7 to 1.5)	0.596	1.3 (0.7 to 2.2)	0.367
Very much (n 1,302)	2.6 (1.8 to 3.8)	0.001	2.6 (1.4 to 4.6)	0.002
Extremely (n 408)	5.7 (3.2 to 10.1)	0.001	4.2 (1.9 to 9.2)	0.001
Age of the personnel				
26–35 (n 848)	ref.			
36–45 (n 1,329)	1.2 (1.0 to 1.5)	0.065	1.6 (1.2 to 2.1)	0.001
46–55 (n 1,246)	1.5 (1.2 to 2.0)	0.001	1.7 (1.3 to 2.3)	0.001
>55 (n 563)	1.6 (1.2 to 2.1)	0.001	1.9 (1.4 to 2.6)	0.001
Q51) educational orientation (public preschool)				
No (n 985)	ref.		ref.	
Yes (n 2,519)	0.9 (0.8 to 1.1)	0.876	1.3 (0.8 to 2.1)	0.253
Q51) educational orientation (predominantly activities outdoors; ur & skur)				
No (n 3,453)	ref.		ref.	
Yes (n 51)	0.5 (0.3 to 0.9)	0.004	0.8 (0.4 to 1.9)	0.633
Q50) Age of the preschool children in the group				
1–3 yrs (n 723)	ref.		ref.	
1–5 yrs (n 1,027)	0.8 (0.6 to 1.0)	0.181	0.7 (0.6 to 0.9)	0.018
3–5 yrs (n 816)	1.3 (1.0 to 1.7)	0.043	1.0 (0.8 to 1.4)	0.825
Q31) Noise sensitivity				
Not at all (n 307)	Ref.		Ref.	
Somewhat (n 1941)	2.2 (1.7 to 2.9)	0.001	2.0 (1.4 to 2.7)	0.001
Rather (n 1437)	4.1 (3.1 to 5.5)	0.001	2.5 (1.7 to 3.5)	0.001
Very (n 291)	7.6 (4.6 to 12.5)	0.001	2.7 (1.4 to 4.9)	0.002

*Adjusted for all items mentioned in the left column

The two questions relating to perceived loudness of the noise exposure at the workplace (Q6, Q7) indicated at crude level a significant influence of the odds of reporting the noise to influence the child’s behavior for all category responses of the questions in relation to never. For the question “Is the sound levels sometimes so loud that you have difficulties hearing what other people say” (Q6), the associations seem to be confounded by other factors. The OR decreased and after adjustment it was not significant for the response alternatives corresponding to loud noise during 75% of the time at work or always/almost always. On the other hand, for the question “How often are you exposed to sound levels so loud that you must raise your voice to be heard?” (Q7), the significantly increased OR remained after full adjustment, with OR ranging from 1.5 to 5.2 for the different response categories.

With regard to noise annoyance (Q5), at crude level, the OR of reporting noise to influence the child’s behavior decreases 50% when the respondent reported noise to be somewhat annoying as compared to not at all. However, this association seemed to be confounded by other factors, as the OR became not significant after adjustment. In the higher response categories, indicating more noise annoyance, the OR increased from 2.6 for “very” annoying to 5.7 for “extremely” annoying as compared to “not at all”. These associations were significant at crude level and were maintained in association and direction after being adjusted for potential confounders, with ORs of 2.6 and 4.2 respectively.

Interestingly age and noise sensitivity of the personnel seemed to be of importance. With increasing age, we found a higher odds ratio to report noise to influence the child’s behavior. The associations remained significant and even higher after adjustments. Similarly, with increasing noise sensitivity a higher odds ratio of reporting noise to influence the child’s behavior was seen. The associations remained significant after adjustments and ranged from 2.0 to 2.7

With regard to the educational orientation of the preschool, crude OR were lower for the outdoor education orientation (Ur & Skur). However, this association was not significant after adjustment, thus probably confounded by other factors.

Finally, the age or age variation of the preschool children seem to be of importance, as adjusted OR for the groups with 1–5 years olds were significantly lower as compared to the younger age groups (1-3years).

### Content analyses

Table 2 reports the number of word stems (denoted by *) or phrases that were found among the free text given as a response to the key question whether preschool noise affected children’s behavior. The derived categories include word stems or phrases representing the same meaning. Only a handful of the answers indicated positive or joyful behavior and being so few they were not considered as a category of its own.

**Table 2:** Content analysis of free text responses to the key question “Do you find that preschool noise affects the behavior of the children?” (Q52), resulting in derived categories, recorded word stems, number of occurrences of each word stem identified in the free text and total occurrences per category.

Derived category	Recorded word stem	Number	Total n per category
To be heard	Loud*	3271	7654
	Voice*	1534	
	Scream*	1157	
	Hear*	412	
	Overhear*	353	
	Increase*	768	
	Yell*	159	
Distracted behavior	Focus*	990	2644
	Concentrat*	926	
	Stress*	728	
Negative internal emotion	Worry* /insecur*	528	605
	Sad*	77	
Exhausted, fatigued	Tire*	434	551
	Irrit*	117	
To crowd	Several*	129	481
	Many*	114	
	Busy*	175	
	Noisy*	63	
Negative external emotional expressions	Conflict*	181	289
	Fight*	51	
	Active*	32	
	Anger/Angry*	25	
Active avoidance	Cover* + ear*	71	128
	Away*	57	
Passive withdrawal	Unwind*	59	85
	Alone*	14	
	Rest*	12	
Difficulties for learning	Learn*	20	31
	Underst*/misunderst*	6	
	Language	5	

* Indicate the truncated word stem searched for in the text.

As can be seen in Table 2, the word “loud” was most frequently reported and was mentioned by more than 3000 personnel. The word loud was included in the category “to be heard” and describes vocal behavior used by the children to be heard, such as scream, yell, use of voice, to be overheard. It was also the category including most words reported by the personnel. Quite a large proportion of the responses dealt with children being unfocused, displaying bot being concentrated and stressful behavior, forming the category “distracted behavior”. The category “negative internal emotions” was reported by around 600 personnel and included the word stems: worry, insecure, and sad. A common description in this category was that some children act out, and become more unfocussed while some get sad and afraid, when there is a lot of noise. Also frequently reported as a description of how children are affected by noise could be related to crowding as noise was being associated to children exiting each other, making them gather, being busy, and noisy. A category of fatigue or exhaustion including words related to tiredness and irritation formed in total 551 expressions. Personnel also reported noise being associated with negative emotional expressions with children acting out, being angry, and having conflicts. Less often but also consistent was reports of active avoidance behavior such as covering the ears, and go away. In addition, passive withdrawal behavior was reported, which possibly indicates a need to unwind, wanting to be left alone, and go quiet. Finally, a category was formed called “learning” which included learning, understanding or misunderstanding, and difficulties with language.

Table 3 shows some examples of the exact phrases given in the free text by preschool teachers, and the corresponding category derived from the content analysis.

**Table 3:** Examples of some of the phrases used by the preschool teachers and the corresponding category. Quotes have been translated from Swedish. The original quotes in Swedish are included within parentheses.

Meaning units	Manifest content	Category
“An increased sound level leads to stress, the children will be louder in order to be seen, to be heard in the group. Other children withdraw if possible” (Id 1276) *(“En ökad ljudnivå ger stress*, *barnen blir mer högljudda för att synas*, *höras i gruppen*, *andra barn går undan om möjlighet finns”)*	Increased sound levels result in more stress.	Distracted behavior
	The children are louder, to be seen and heard.	To be heard
	Other children withdraw.	Passive withdrawal
”They get stressed, have difficulties to concentrate, go away, scream louder, more conflicts, difficult to unwind” (Id 386) *(“De blir stressade*, *svårt att koncentrera sig*, *går undan*, *skriker högre*, *mer konflikter*, *svårt att koppla av”)*	Children get stressed and have difficulties concentrating.	Distracted behavior
	Children go away.	Active avoidance
	Children scream louder.	To be heard
	There are more conflicts.	Negative external emotion
	Children have difficulties to wind down.	Passive withdrawal
” Some children get worried and cry, many raise the voice and scream to each other..” (Id 150) *(*”*vissa barn blir oroliga och gråter*, *många höjer sina röster och skriker till varandra*..*”)*	Children get worried and cry	Negative internal emotion
“They get loud and have difficulties concentrating. Very tired in the afternoon” (Id 850) *(“De blir högljudda och de har svårt att koncentrera sig*. *Blir väldigt trötta på eftermiddagen*.*”)*	Children become loud.	To be heard
	Children have difficulties concentrating.	Distracted behavior
	Children become very tired over the course of the day.	Exhaustion, fatigued
”The children become stressed by the noise and exite each other (Id 556) "Barnen blir stressade av buller och”jagar” upp varandra	Stressed children exite other children	To crowd
“Call out more and louder. Do not wait for e.g. an answer. Several children have difficulties with language and sounds” (Id 854) *(”Ropar mera och högre*. *Väntar inte in t ex svar osv*. *Svårigheter med språk och ljud har flera barn*.*”)*	Children yell more and louder.	To be heard
	Do not wait for an answer.	Distracted behavior
	Many children have difficulties with sounds and language.	Difficulties for learning
”Yell to each other and fight more often now, kick (hit) each other often, have difficulties listening to each other” (Id 847) *(”Skriker till varandra och slåss mera än tidigare*. *Sparkar varandra ofta*, *svårt att lyssna på varandra*.*”)*	Children yell.	To be heard
	Children act out more often and have more conflicts.	Negative external emotion
	Children don’t pay attention to other children.	Distracted behavior
”Children complain of headache and I can see that the motivation to learn and to learn something new decrease when the sound level increase” (Id 5584) *(“Barnen klagar på huvudvärk och jag kan se att motivationen att lära sig*, *att lära något nytt sjunker då ljudnivån stiger”)*	Children get headache.	Exhaustion, Fatigued
	Motivation to learn decrease.	Difficulties for learning
	Motivation to learn new things decrease.	Difficulties for learning
“Tired, whining, irritating wants to hide/go away” (Id 612) *(“Trötta*, *gnälliga irriterade*, *vill gå undan”)*	Children get fatigued.	Exhaustion, fatigued
	Children hide.	Passive withdrawal

The sensitivity analysis performed separately by one of the authors confirmed the previously derived categories and none of the 50 random samples included reports of positive effects of noise in children’s behavior. From the random examples, it became even more obvious that some children crowd, but many try to avoid noisy situations.

## Discussion

The content analysis of the teachers’ perspective on how preschool noise affects children’s behavior revealed findings that to the best of our knowledge have not previously been described. First, it became apparent that a vast majority of the personnel included in this study considered that children’s behavior was affected rather or very much by preschool noise. Second, the content analysis revealed nine separate categories of noise related behavior, with the most common being: to be heard, to be distracted/stressed, insecure, exhausted/fatigued, and to crowd. A functional manifestation of such behavior could result in maladaptive coping patterns, which in the long run could be disruptive for health [11]. Such behavior patterns could also hamper the child’s development of social skills and language learning [12].

### Primary findings

Vocal behavior leading to loud sounds, screams, yelling with the intention to be heard, was the most often adopted behavior by the children when there was noise. It cannot be excluded that this description of the children’s behavior is confounded by the observation pattern of the personnel. However, in the context of the very high noise levels frequently found at preschools, ranging from 80–85 dB LAeq—this behavior is comprehensible. The speech level of a relaxed speaking person is usually in the range of 55–60 dBA at one meters distance. With increasing background level adults tend to increase their voice. It is highly probable that this also applies to children. This phenomenon was discovered 1911 and is referred to as the Lombard effect. If the surrounding noise is in the range of 80–85 dBLAeq, a child would have to scream with more or less full effort to make him/herself being heard, and our results show that this is also noted by the personnel under noisy conditions. It is possible that after a longer period in noisy settings children maintain this way of coping, also when the background noise level decreases. The behavior was verified in another study where about 40% of parents responded that their preschool children often or always, talked with a loud voice also at home [7]. Interestingly, the content analyses were confirmed by the quantitative analyses where we first see a clear trend in the OR by the exposure i.e. as the occurrence of loud noise exposure increase, the OR of the outcome also increases and becomes more significant. Second, the association with increased levels of noise exposure seems to be unbiased even when considering other factors, such as noise annoyance.

The vocal behavior and the negative external emotional expressions of acting out and aggressive behavior may also be a way of gaining control over a situation that is experienced by the child as being out of control or chaotic. Studies on the influence of the number of children per household and household chaos have shown that children respond to the accompanying increase in noise levels from more voices by raising their own volume [13]. Aggression and acting out can be considered as an effect of household chaos, but also as a determinant of chaos. It is plausible to assume that the increasing number of children in the preschool groups plays a role for the noise level and some of the effects here reported. In the 1970s, the recommended number was 10–12 children per group [14, 15], while the average was 16.9 children per group in 2013 [16]. Our study could however not verify or reject this assumption. The question on number of children in the group was not considered valid, as it was possible that the personnel misinterpreted it to mean the whole school. Kihlbom, Lidholt and Niss emphasize that the number of children per group and the number of personnel are the two most important structural factors for a high quality in the preschool [17]. These factors are according to them particularly important for children’s language development, the interplay and attachment between children and grown-ups, children’s development of identity, noise, stress, and conflicts. In Sweden, many preschools were built 30 years ago, when the number of children in the preschools was fewer, making them too small for today’s larger groups. When the number of children is too large for the space provided stress may arise both among the personnel and among the children. For example, planned activities become difficult and children cannot engage in undisturbed play [18]. While group size seem to be important for learning and child-development there is remarkably little research of its impact on noise and noise related behavior. Further studies are clearly needed.

The personnel describes that noise makes children more unfocused and less concentrated which could aggravate the perceived chaos. The meaning formulated in the model: “Living with own uncontrollability of sounds and noise” based on the child perspective was put forward in an earlier study [6]. In situations where children perceive no control, it may be natural to try to regain control by some type of coping strategy. According to Wadsworth, children progress from the infant coping behavior including crying and seeking physical comfort towards actively seeking help and avoidance of sources of stress during the toddler and preschool age [11]. In this study, the personnel did not mention seeking help. However, avoidance behavior, withdrawal, and wanting to be left alone was mentioned. As children have less ability to anticipate and understand stressors in general [19], there is a risk that children functionally adapt to noise by maladaptive coping behavior. Although it was not reported as a highly frequent behavior in this study, we have earlier found when asking children directly, that more than 70% adopted some sort of coping mechanisms, such as going away and covering the ears [7].

Apart from studies on noise, the crowding literature is relevant for understanding the mechanisms behind this behavior, for an overview see Creed [20]. Children adapt to crowding by using withdrawal as a coping mechanism, less engagement in activities and less play with other children [13]. Although crowding, like environmental noise, has been found to effect reading and word identification, the evidence has been mixed on how it influences language acquisition and vocabulary [13]. Evidence from schoolchildren shows that they cope with chronic noise in their environment by ignoring or disregarding auditory inputs [13, 21]. An unfortunate consequence of this is that also important speech is tuned out, increasing the risk for reduced writing and reading abilities [13]. Non-native language speaking children and children with language disorder and hearing impairments may be particularly at risk in poor sound environments.

This might also be the case for children with behavioral problems. Stansfeld et al have reported an increased hyperactivity score on the Strength and Difficulty Questionnaire (SDQ) associated with air traffic noise exposure at home in children 9–11 years old [22]. The hyperactivity results may indicate that high aircraft noise exposure exacerbates hyperactivity symptoms in children already at risk. Also, the way teachers interact with children under noisy circumstances can affect children’s development. Research has shown that teachers are more fatigued, less patient towards the children, and more prone to annoyance when they are chronically exposed to noise [23].

Educational orientation was not found to strongly influence if the personnel considered noise to affect children’s behavior. The most obvious reason for this is that the noise levels did not differ much between preschools of different education orientation or that noise regardless of educational orientation affected children’s behavior. Further studies are needed to understand how didactics effect noise and the children’s behavioral response to the preschool sound environment.

Age of the preschool teachers affected the reporting on how noise affected children’s behavior: the higher the personnel age, the higher the OR of reporting an effect. It is not clear whether this is related to individual factors such as a higher awareness related to age-related hearing impairment, inclusive a sensitization to noise or professional experience. Noise sensitivity also affected the reporting, with a higher noise sensitivity being associated to a higher OR of reporting children being affected by noise. Noise sensitivity refers to the internal states of an individual (physiological, psychological, or life-style determined), which increases the degree of reactivity to noise in general, hence, the association between noise sensitivity and reporting children being affected is plausible [24]. Of further interest is also to study how the behavior of preschool teachers affects children’s behavior, such as teachers raising their voice.

### Methodological considerations

This is one of the first studies addressing the effect of noise on preschool children from a teacher perspective. Since so little is known about the reaction of young children to environmental noise and noise in general, a qualitative approach is a suitable way of developing a nuanced description. This allows us to better understand different behavior that children adopt in a noisy setting. The content analysis based on the teachers own free text responses enabled us to derive conclusions with high ecological validity. The results are based on female preschool teachers as only a very small percentage of men work as preschool teachers and consequently the sample of men that originally answered was very small (3%). It is possible that answers from male preschool teachers had resulted in additional categories. However, this is not possible to conclude on from this study. Furthermore, adults may not be the best ones to judge children’s noise-induced level of discomfort and stress as adults’ hearing and perception of sounds seem to differ with regard to small children. Ongoing studies therefore seek to obtain further information on children’s perception of their sound environment using validated questionnaire [7].

Being a cross-sectional study, the direction of the associations cannot be determined. It is possible that the children’s behavior primarily drives the noise situation in preschools, as indicated in the open questions to the teachers in which they describe noise being associated with children crowding, and being busy and loud. Nevertheless, the majority of the teacher’s answers indicated that noise might have consequences for children’s behavior in ways that have bearing in the psychological literature, as described above.

## Conclusions

Content analyses of teachers reporting of preschool children´s behavior in noisy settings informed how noise affects children’ behavior in a variety of ways. The most commonly described categories were children´s need to be heard, children being distracted, emotionally affected, and tended to show crowding, avoidance, withdrawal, and exhaustion. The quantitative analyses confirmed the association between estimated loudness and noise annoyance at preschool and affirmative reporting on noise affecting the children´s behavior. Age of the personnel, with the youngest, reporting noise related behavior less often, and age distribution of the class with 1–5 years old being less affected, were also indicated. Future studies need to address the way in which children cope with noise and its long-term consequences for health and learning. Also of interest is the influence of teachers’ behavior on the child in noisy situations.
